# Heat impact during laser ablation extraction of mineralised tissue micropillars

**DOI:** 10.1038/s41598-021-89181-9

**Published:** 2021-05-26

**Authors:** Samuel McPhee, Alexander Groetsch, Jonathan D. Shephard, Uwe Wolfram

**Affiliations:** 1 Institute of Mechanical, Process and Energy Engineering, School of Engineering and Physical Sciences, Heriot-Watt University, Edinburgh, UK; 2 Institute of Photonics and Quantum Sciences, School of Engineering and Physical Sciences, Heriot-Watt University, Edinburgh, UK; 3 Present Address: Swiss Federal Laboratories for Materials Science and Technology, Laboratory for Mechanics of Materials and Nanostructures, Thun, Switzerland

**Keywords:** Laser material processing, Biomedical engineering

## Abstract

The underlying constraint of ultrashort pulsed laser ablation in both the clinical and micromachining setting is the uncertainty regarding the impact on the composition of material surrounding the ablated region. A heat model representing the laser-tissue interaction was implemented into a finite element suite to assess the cumulative temperature response of bone during ultrashort pulsed laser ablation. As an example, we focus on the extraction of mineralised collagen fibre micropillars. Laser induced heating can cause denaturation of the collagen, resulting in ultrastructural loss which could affect mechanical testing results. Laser parameters were taken from a used micropillar extraction protocol. The laser scanning pattern consisted of 4085 pulses, with a final radial pass being 22 $\upmu {\text {m}}$ away from the micropillar. The micropillar temperature was elevated to 70.58 $^{\circ }{\text {C}}$, remaining 79.42 $^{\circ }{\text {C}}$ lower than that of which we interpret as an onset for denaturation. We verified the results by means of Raman microscopy and Energy Dispersive X-ray Microanalysis and found the laser-material interaction had no effect on the collagen molecules or mineral nanocrystals that constitute the micropillars. We, thus, show that ultrashort pulsed laser ablation is a safe and viable tool to fabricate bone specimens for mechanical testing at the micro- and nanoscale and we provide a computational model to efficiently assess this.

## Introduction

Bone related diseases such as cancers, osteoporosis and osteoarthritis pose significant personal and socio-economic burdens. Bone cancers, for example, whether primary or metastasised, increase morbidity and mortality by inducing chronic bone pain and increasing the risk of skeletal events such as pathological fractures^1,2^. Laser ablation can be used as a minimally invasive palliative treatment to mitigate morbidity. Clinical procedures that utilise laser ablation often use continuous wave infrared light to photocoagulate malignant tissue in situ^3–5^. However, the temperature exposure of surrounding healthy tissue and the uncertainty regarding the exact elevation has suppressed widespread acceptance of this technique^6^.

In contrast to continuous wave sources, ultrashort pulsed laser ablation is a minimally-invasive resection technique with viability for use in orthopaedic surgery^7–9^. The distinctive ultrashort temporal profile (<10 ps) and high spatial resolution of a pulse, generates intensities ($>10^{14}\;{{\text {W/cm}}}^{2}$) sufficient to induce localised non-linear absorption of incident photons through electron-photon coupling. In bone tissue, this non-linearity results in the rapid generation of free electrons which subsequently exhibit optical properties similar to that of conduction electrons in metals. This ionisation limits further photon penetration to a well-defined thin surface layer, where electrons are excited up to tens of eV^10^. Nonthermal ablation mechanisms such as Coulomb explosion and electrostatic ablation then dominate over melting and evaporation. This localised energy deposition and subsequent ablation occurs in a period prior to considerable energy exchange to the bulk tissue, which remains at a temperature close to ambient^11,12^. The heat affected zone of a single pulse is minimal, however, thousands of pulses are required to mitigate low ablation rates during, for example, orthopaedic hole drilling^13^. The cumulative heat deposition here can effect the physio-chemical and microstructural composition, as well as the cellular viability of tissue surrounding the ablated region. The cellular viability is of particular importance during targeted removal in the order of micrometres, for example when ablating close to nerve tissue. Therefore, it is important to quantify the temperature response of bone tissue during a complete scanning laser ablation regime for such a removal.

The thermal response of bone tissue can be divided into a cellular and an extracellular response. At the cellular level, temperatures in excess of $47\,{^{\circ }}{\text {C}}$ for 60 s can induce bone resorption, cell death and vascular cessation^14^. The extracellular matrix response is governed by bones major constituent components, which are; collagen (30–40 wt%), mineral (50–60 wt%), and water (10–20 wt%)^15–17^. The thermal response of the mineral content can be largely overlooked, as phase transition occurs at a temperature ($>800\,{^{\circ }}{\text {C}}$) an order of magnitude higher than that of collagen^18^. The collagen content in bone comprises of 90–95% Type I collagen^19,20^. Excessive heating can cause denaturation, where stabilising inter- and intramolecular covalent cross-links are ruptured, leading to irreversible changes in conformation^21^. Several experimental methods have been used to investigate the thermomechanical response of collagen, including differential scanning calorimetry and microthermal atomic force microscopy^22–26^. Tissue hydration has been shown to be a major determinant of denaturation temperature. Miles et al.^27^ demonstrated that reducing the volume of interfibrillar fluid by dehydration increases the thermal stability of collagen. This increase in stability by dehydration is evidenced by the observations of several authors, where heating of hydrated samples yields denaturation temperatures in the range of 50–70 $^{\circ }{\text {C}}$^25,26,28,29^, while dehydrated heating yields temperatures in the range of 140–160 $^{\circ }{\text {C}}$^26,30^. Like the cellular response, collagen denaturation does not only depend on the magnitude of temperature, but also on the exposure time. Isothermal exposure tests have shown that reversible localised unfolding occurs prior to irreversible changes in conformation^31^. Chen et al.^23^ investigated fibril shrinkage behaviour through isothermal temperature jump tests and observed, for example, at 70 $^{\circ }{\text {C}}$, fibril shrinkage is reversible at exposure times less than $\sim 1.4$ s. High pulse repetition frequency protocols that are used to overcome poor ablation rates in orthopaedic hole drilling can deliver thousands of pulses in a 1.4 s time frame. Monitoring and understanding the permissible temperature exposure with respect to time and magnitude will allow for specific laser ablation treatment protocols to be optimised for increased bone removal rates.

The need to quantify the thermal response of bone during ultrashort pulsed laser ablation is not limited to in vivo surgical applications. The micrometre precision has been utilised for the extraction of micrometre-sized mechanical test specimens^32,33^. One example, micropillar extraction, requires ablation of an annulus shaped channel to isolate a pillar that protrudes from the bulk tissue. Subsequent micro-compression allows for the direct assessment of the micro- and nanoscale mechanical behaviour of bone^33–35^. Thermally induced denaturation of the collagen that comprise these pillars may invalidate testing results as the composition will no longer represent that of the unprocessed bone. We herein focus on a laser protocol used by Groetsch et al.^33^ for extraction of micropillars. These comprised of mineralised collagen fibres, which are the fundamental structural unit that underpins the microscale of bones hierarchical composition^36^. Groetsch et al.^33^ used the mineralised turkey leg tendon as a model system for bone as they both exhibit the same hierarchical setup at the microscale^37–39^. The used protocol involved dehydration of samples after dissection, whereas in vivo surgical procedures operate under hydrated physiological conditions. This dehydrated status of Groetsch et al.^33^ samples allows for a higher permissible operating temperature than orthopaedic procedures. We deem 150 $^{\circ }{\text {C}}$, irrespective of any exposure time, as a maximum temperature prior to denaturation as Bozec and Odlyha^26^ report this as the temperature at which intramolecular hydrogen bonded water evaporates in dry collagen fibrils. This bonding stabilises the ultrastructure of the collagen helix, and it can be inferred that heat induced loss will allow for fragmentation at lower mechanical loads.

Verifying that the laser-tissue interaction has no detrimental effect on bone composition is crucially important to safely produce micropillars for investigating the micro- and nanoscale mechanical behaviour of bone. Temperature monitoring devices lack the resolution required to quantify the temperature at the micro- and nanoscale. Therefore, the aim of this paper was to (1) implement a heat model representing the laser-tissue interaction into a finite element suite to quantify the temperature elevation during micropillar extraction using picosecond laser ablation; (2) use the model to evaluate the existing ablation process employed by Groetsch et al.^33^ for micropillar extraction; (3) use post-production compositional analysis to qualitatively asses denaturation; and (4) use the model to evaluate minimal pillar sizes through laser ablation only.

## Materials and methods

### Picosecond ablation of pre-pillars

**Figure 1:**
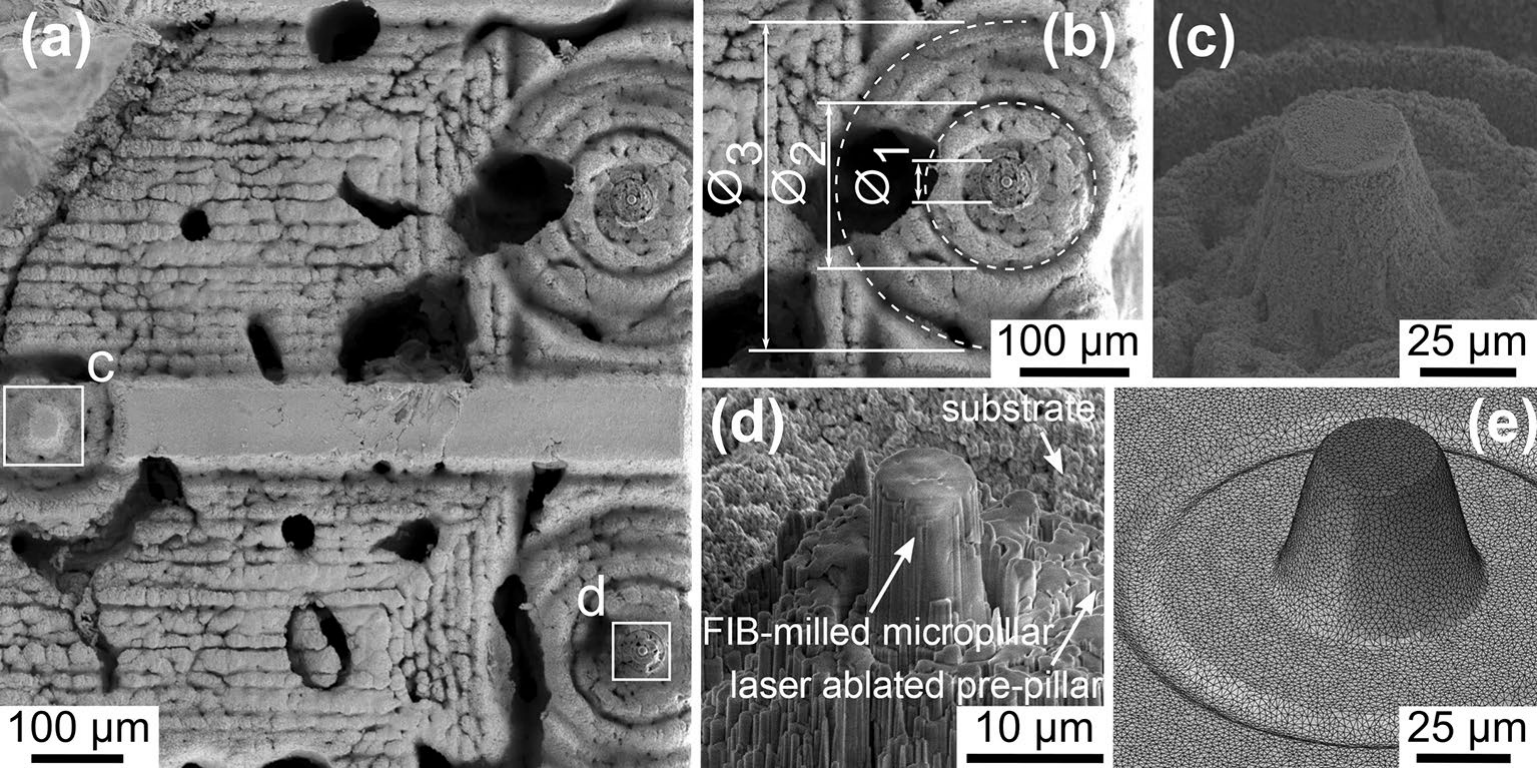
Micropillar extraction overview. (**a**) SEM image of a the final processed MTLT sample, with two micropillars present^41^. (**b**) area ablated by spiral inbound anticlockwise hatch patterns, where $\varnothing _{1} = 32.85 \pm 0.91\;\upmu {\text {m}}$, $\varnothing _{2} = 150\;\upmu {\text {m}}$, $\varnothing _{3} = 300\;\upmu {\text {m}}$^41^ (**c**) SEM image of a pre-pillar^41^. (**d**) SEM image of a FIB milled micropillar^33^. (**e**) finite element mesh of the pre-pillar. The circular groove here corresponds to $\varnothing _{2}$ in (**b**); where the scanning patterns of the outer and inner spiral hatch patterns overlap.

Groetsch et al.^33^ fabricated pre-pillars (Fig. 1c) using a thin-disk TruMicro 5250-3C laser (Trumpf, Germany) which delivered pulses with a full width at half maximum duration of 6 ps at a pulse repetition frequency (PRF) of 1 kHz, operating at a wavelength of $\lambda =515$ nm in TEM_00_ (M$^{2} \sim 1.33$). The laser was operated with an average power of 12.125 mW, corresponding to a pulse energy of 12.125 ${\upmu }$J. An f-theta lens with a focal length of 160 mm resulted in a beam waist radius of $\omega _0=10$ $\upmu {\text {m}}$, measured at 1/*e*^2^. A galvanometer scan head was used to scan the focused spot across the tissue surface at a speed of 2 mm/s, which with a 1 kHz repetition frequency, corresponds to a 90% beam overlap.

Prior to laser ablation, mineralised turkey leg tendon (MTLT) samples were dissected by scalpel and diamond band saw (Exakt, Norderstedt, Reichert-Jung) before being glued into an aluminium sample holder. The result was an exposed cylinder of tendon of 1.5 mm in diameter and 2 mm in height. The free surface was ultramilled (Polycut E, Reichert-Jung, Germany) to provide a plane surface for compression tests. For the laser ablation extraction, a spiral inbound anticlockwise hatch pattern was centred around each fibre, from an outside diameter of 300 $\upmu {\text {m}}$ inward to 150 $\upmu {\text {m}}$ (Fig. 1b). Each successive radial pass reduced in radius by 2 $\upmu {\text {m}}$, consistent with the 90% beam overlap. A final pattern with consistent parameters then scanned from 150 $\upmu {\text {m}}$ inward to 50 $\upmu {\text {m}}$ (Fig. 1b). The heights and diameters of the pre-pillars were quantified by means of three-dimensional surface topography profiles using non-destructive focus variation microscopy (Alicona Infinite Focus, Austria)^40^ as well as SEM (Quanta 3D FEG, FEI, USA). The resultant pre-pillars were $32.85 \pm 0.91\;\upmu {\text {m}}$ in diameter and $50.01 \pm 4.04\;\upmu {\text {m}}$ in height^33^. Inbound rectangle hatch patterns were used to ablate substrate far from each fibre to produce channels to allow for X-ray diffraction acquisition during compression testing (Fig. 1a). Focused ion beam milling (Dual FIB-SEM Quanta 3D FEG, FEI, USA) was then used as a final cut to extract micropillars (Fig. 1d) of $6.08 \pm 0.83\;\upmu {\text {m}}$ in diameter and $12.44 \pm 1.78\;\upmu {\text {m}}$ in height. For specific details regarding the focused ion beam milling process see Groetsch et al.^33^.

### Geometric and mesh configuration

A surface profile representing the MTLT after each laser pulse in the spiral inbound anticlockwise hatch pattern was developed. This represented ablation, where after each pulse, a volume was removed depending on its respective pulse location. This was done through interpretation of the pre-pillar measurements and the known laser parameters. Firstly, the threshold fluence of the MTLT was estimated assuming that the spatial profile of the focal spot was a Gaussian^32^:

1 $$\begin{aligned} F_{th} = 2\frac{E_p}{\pi \, \omega_0^2}\,\exp \left( \frac{-2r_{th}^2}{\omega_0^2}\right) , \end{aligned}$$

where $F_{th}$ denotes the threshold fluence, $E_p$ the pulse energy, $\omega _0$ the beam radius (measured at $1/e^{2}$) and $r_{th}$ the threshold radius; beyond which no ablation occurs. This threshold radius was interpreted as the difference between the final programmed radius of the scanning pattern and the measured pre-pillar surface radius.

**Figure 2:**
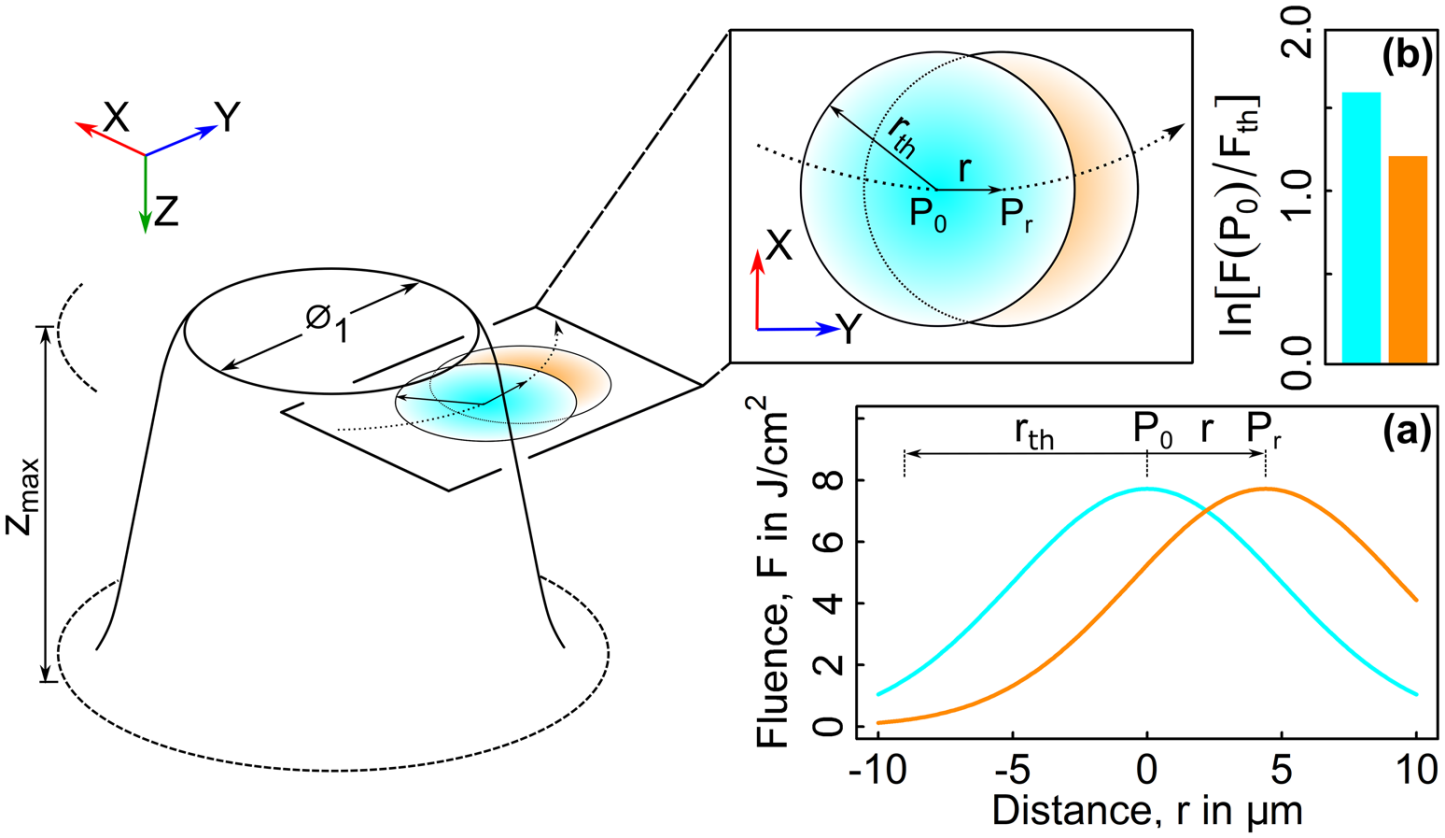
Schematic of the laser ablated pre-pillar. The circular dashed line that runs through the beam axis of the two presented pulses (orange and cyan circles) is the final radial scanning pass of $25\;\upmu {\text {m}}$. ${r}_{th}$ is the threshold radius and r is the arbitrary distance between point ${\text {P}}_0$ and ${\text {P}}_r$. $\varnothing _{1}$ and *z*_*max*_ are the surface diameter and pillar height respectively. Graph (**a**) represent the fluence incident on position P_0_ (Eq. 1) and graph (**b**) shows the dimensionless ratio of depth (Eq. 2) for both pulses (orange and cyan).

The threshold fluence was then used to find an effective penetration depth of the laser. Here, an arbitrary pulse, P_0_ and its respective position is selected within the scanning pattern, where the pulse location corresponds to its Gaussian beam axis. A second pulse, P_r_, at distance r will induce a fluence at position P_0_. All pulses in the scanning pattern that are located within the threshold radius about P_0_ will add to the ablated depth at position P_0_ (See Fig. 2). An approximate penetration depth that induces ablation can be derived from the sum of the logarithmic dependence of ablated depth per pulse^42^:

2 $$\begin{aligned} \delta p_{eff} = {\frac{z_{max}}{\sum \nolimits _{i=1}^{N_p}\ln \left( \frac{F_{i}}{F_{th}}\right) }}, \end{aligned}$$

where $\delta p_{eff}$ is the effective penetration depth. $N_p$ denotes the number of pulses with beam axis positions within the threshold radius about point P_0_. $z_{max}$ is the final cumulative ablated axial depth at P_0_. $F_{i}$ is the fluence incident on P_0_ for each respective pulse that is within the threshold radius.

The final spiral inbound anticlockwise hatch pattern from a diameter of $150\;\upmu {\text {m}}$ inward to $50\;\upmu {\text {m}}$ (Fig. 1b) consisted of 4085 pulses. A uniform x–y grid of nodes was generated in MATLAB (MATLAB R2019a, MathWorks) with all nodes being assigned an initial depth, z, of $0\;\upmu {\text {m}}$ to represent the ultramilled surface of the MTLT. For a single pulse, the nodes that were within the threshold radius about the x and y coordinates of its Gaussian beam axis were displaced to represent the ablated volume using:

3 $$\begin{aligned} \Delta z\left( r,z\right) = \ln {\left( \frac{F\left( r,z\right) }{F_{th}}\right) }\,\delta p_{eff} \end{aligned}$$

here $\Delta z$ is the specific axial displacement of each node within the threshold radius. $F\left( r,z\right)$ is the magnitude of fluence at each node given by:

4 $$\begin{aligned} F\left( r,z\right) = 2\frac{E_p}{\pi \,\omega^2\left( z\right) }\,\exp \left( \frac{-2r^2}{\omega^2\left( z\right) }\right) \end{aligned}$$

here *r* is the radial distance between the pulse specific Gaussian beam axis and a node within its threshold radius. $\omega \left( z\right)$ accounts for radial propagation of the Gaussian beam as a function of axial depth, given by^43^:

5 $$\begin{aligned} \omega \left( z\right) = \omega _0\left[ 1+\left( \frac{\left( z-z_0\right) \,\lambda M^2}{\pi \,\omega _0^2}\right) ^2\right] ^{0.5} \end{aligned}$$

here $\lambda$ is the wavelength of light. $z_0$ is the axial depth of focus. The beam quality factor, $M^2$, was 1.33. All 4085 pulses in the scanning pattern were resolved and the resultant nodal surface profiles after each pulse were triangulated. The profile resulting from the final radial pass of $25\;\upmu {\text {m}}$ was $31.94\;\upmu {\text {m}}$ in diameter and $49.8\;\upmu {\text {m}}$ in depth (Fig. 2).

The triangulated surface profiles were extruded in MATLAB to produce bounded STL shells representative of the volume of MTLT. The STL shells were then resampled in PyMesh^44^ such that the maximum tri-face corner angle of any surface triangle was $120^{\circ }$. The STL shells were volumetrically meshed with linear tetrahedral elements in Gmsh^45^. Finally the elements were converted to quadratic tetrahedral elements in MATLAB through the addition of midside nodes. The above process generated a directory of 4085 pulse specific geometric profiles for which a heat transfer analysis could be applied.

### Heat model

In this study we were interested in the cumulative temperature elevation induced by a scanning pattern and not the specific phase change mechanism within the ablated volume of each incident pulse. For this reason, we used the aforementioned effective absorption coefficient to approximate the removal after each pulse. In ultrashort pulse ($<10$ ps) irradiation of dielectrics, the laser-tissue interaction can be explained in three stages. First the laser energy is absorbed through photon–electron coupling. In dielectrics, electron heating is mediated by non-linear absorption processes that are required to first generate free electrons, such as multiphoton and tunneling ionization. Free electron generation causes the material exhibit metallic like optical properties which localises energy deposition to a thin surface layer^12,46^. These highly excited electrons then exchange energy to the ambient lattice through electron–phonon coupling. Finally energy diffuses into the bulk material through phonon–phonon collision. The electron–phonon coupling can be described by the two temperature model where two general heat equations are coupled by an electron–phonon coupling parameter^47^:

6 $$\begin{aligned} C_e\frac{\partial T_e}{\partial t}= & {} \nabla \cdot k_e\nabla T_e - \gamma \left( T_e-T_l\right) + Q\left( x,y,z,t\right) \end{aligned}$$

7 $$\begin{aligned} C_l\frac{\partial T_l}{\partial t}= & {} \nabla \cdot k_l\nabla T_l + \gamma \left( T_e-T_l\right) \end{aligned}$$

here $C_e$, $C_l$, and $k_e$, $k_l$ are the electron and lattice heat capacity and conductivity, respectively. $Q\left( x,y,z,t\right)$ is the energy absorption rate and $\gamma$ is the electron–phonon coupling parameter. We are interested in the cumulative temperature elevation within the micropillar after a time in the order of ms. Inspection of the surface morphology revealed that the chosen process parameters resulted in a negligible degree of melting and recast (See Fig. S1 in “Supplementary Materials”). Therefore, we treat the ablated volume as an isolated mechanism, allowing us to remove the respected ablated volume at an arbitrary time which we chose at the end of the pulse. Further to this, we assume there is negligible energy exchange to the tissue bulk from the ablated volume^10^. A second assumption is that outside of the threshold radius, the intensity does not induce non-linear absorption such that the optical properties remain constant; and and finally, thermalisation between the electron and lattice systems is instantaneous $\left( T_e = T_l = T\right)$ so that the coupled heat equations can be simplified to a single general heat equation as follows:

8 $$\begin{aligned} \left( C_e + C_l\right) \frac{\partial T}{\partial t} = \nabla \cdot \left( k_e + k_l\right) \nabla T + Q\left( x,y,z,t\right) . \end{aligned}$$

We approximate that the sum of the electron and lattice heat capacities to be equal to the bulk heat capacity where $\left( C_e + C_l\right) = \rho c_p$^48^. Here $\rho$ is density and $c_p$ is specific heat capacity. The thermal conductivity for the electron and lattice domains become $\left( k_e + k_l \rightarrow k\right)$ where *k* is the bulk material thermal conductivity, such that the heat addition is spatially ideal. Implementing these, Eq. (8) becomes:

9 $$\begin{aligned} \rho c_p\frac{\partial T}{\partial t} = \nabla \cdot k\nabla T + Q\left( x,y,z,t\right) . \end{aligned}$$

The energy absorption rate, *Q*, in Eq. (9) can be described by the product of a spatial and temporal term where $Q\left( x,y,z,t\right) = S\left( x, y, z\right) T\left( t\right)$. The spatial term $S\left( x, y, z\right)$, is given by^47^:

10 $$\begin{aligned} S\left( x, y, z\right) =k\,\mu _{eff} \,(1-R) \, F_0 \, \frac{\omega _0^2}{\omega ^2\left( z\right) }\,\exp {\left( \frac{-2\left( \left( x-x_0\right) ^2+\left( y-y_0\right) ^2\right) }{\omega ^2\left( z\right) }+ \mu _{eff}(z_s-z) \right) } \end{aligned}$$

here $\left( 1-R\right)$ is the absorptivity where *R* is the reflectivity of the target surface. $F_0$ is the magnitude of the fluence where $F\left( x = 0,\,y = 0,\,z = 0\right)$. $x_0$ and $y_0$ correspond to the *x* and *y* coordinates of the Gaussian beam axis relative to the central axis of the inbound anticlockwise hatch pattern. $z_s$ is the surface depth at any *x* and *y* position, such that the surface intensity is independent of surface depth, while remaining relative to the focal depth. The mineral content in bone results in the domination of light scattering over absorption. To account for an increased fluence rate proximal to the material surface due to back-scattered reflectance, we use an effective attenuation coefficient, $\mu _{eff}$, and a scalar back-scatter coefficient, *k*, where^49,50^:

11 $$\begin{aligned} \mu _{eff} = \sqrt{3\mu _a\left[ \mu _a + \mu _s'\right] }, \end{aligned}$$

$\mu _a$ and $\mu _s'$ are the absorption and reduced scattering coefficients respectively. The temporal intensity, $T\left( t\right)$, is given by^47^:

12 $$\begin{aligned} T\left( t\right) = \sqrt{\frac{4\ln {(2)}}{\pi \,\tau _p^2}}\,\exp \left( {-4\ln {2}\left( \frac{t-2\tau _p}{\tau _p}\right) ^2}\right) , \end{aligned}$$

where $\tau _p$ is the full width at half maximum (FWHM) pulse duration. Upon initial irradiance, free-electrons generated through photoionisation begin to exhibit optical properties similar to conduction electrons of metals. This subsequently shortens optical penetration and cumulative energy deposition to a finite depth^12^. Further excitation towards critical electron density sees a rapid increase in reflectivity that has a strong shaping effect on the temporal intensity $T\left( t\right)$^10^. To account for these non-linearities, which prevent heating in material beneath the ablated region, we make the first order approximation:

13 $$\begin{aligned} Q\left( x,y,z,t\right) = {\left\{ \begin{array}{ll} S\left( x, y, z\right) T\left( t\right) , &{} {\text {if }} t < t_0 \\ S\left( x, y, z\right) T\left( t\right) , &{} {\text {if }} t \ge t_0\ {\text {and }} \sqrt{\left( x-x_0\right) ^2 + \left( y-y_0\right) ^2} > r_{th}, \\ 0, &{} {\text {if }} t \ge t_0\ {\text {and }} \sqrt{\left( x-x_0\right) ^2 + \left( y-y_0\right) ^2} \le r_{th} \end{array}\right. } \end{aligned}$$

where $t_0$ is taken as the time at which Eq. (12) is maximum, minus half the FWHM pulse duration. A visual representation of these spatial and temporal functions can be found in Video S1 in the “Supplementary Materials”. All modelling parameters are summarised in Table 1.

### Numerical modelling

The heat model described above was implemented into a finite element modelling suite (Abaqus 2016a, Dassault Systemes) to simulate the laser-tissue interaction. The model consisted of the aforementioned directory of predefined meshes that were each representative of pulse specific surface profiles. The constituent elements of each mesh were 10-node DC3D10 heat transfer tetrahedrons and were assigned the thermophysical properties required to implement Eq. (9): density, specific heat and thermal conductivity (Table 1). Groetsch et al.^33^ used MTLT as a model system for bone in their studies to facilitate the extraction of micropillars from individual mineralised collagen fibres for their micro- and nanomechanical testing. At the microscale, the MTLT exhibits the same hierarchical setup as bone^37–39^. For this reason, we used published values for cortical bone. The studies from which these parameters were obtained were observed at the macroscopic length scale. We used these as no data was available for the mineralised collagen fibre level. As cortical bone exhibits a low macroporosity (5–15%)^17^, we assume these properties scale down to the micrometre length scale. Further to this, Davidson and James^51^ report that heat transfer in cortical bone can be regarded as isotropic. Therefore, the general heat equation that we modelled was:

14 $$\begin{aligned} \rho c_p\frac{\partial T}{\partial t} = k\nabla ^2T + Q\left( x,y,z,t\right) . \end{aligned}$$

The spatial term, $S\left( x, y, z\right)$, of the energy absorption rate (Eq. 10) was discretised such that each element was assigned a magnitude of flux depending on the coordinates of its centroid. Each mesh was partitioned into two element sets, where element assignments where based on the geometric condition in Eq. (13). Two temporal amplitudes representing $T\left( t\right)$ were then assigned to each element set depending on the temporal condition in Eq. (13). An initial boundary condition at the start of the simulations was $T\left( x, y, z, 0\right) = T_{amb}$. The target (free) surface was assigned a surface film condition of *h*_*a*_ = 10 W/m^2^ K to represent free convection with air^52^. The remaining five surfaces that would be embedded were assigned a surface film condition of *h* = 270 W/m^2^ K such that any element face on these surfaces were subject to one dimensional heat transfer to an ambient sink 2 mm away from its respective surface (*h* = $k/0.002~(T - T_{amb})$).

There were two characteristic time steps for each simulated pulse in the scanning pattern. The first was the laser heating step and was relative in length to the pulse duration at 20 ps. During this step the energy absorption rate was applied. Following this step the next mesh was initiated which represented the surface profile after the ablated volume was removed. The temperature distribution at $t=20$ ps was then mapped onto the new mesh. The second step was a relaxation period between pulses of 1 ms. During this step the dense temperature field about the Gaussian beam axis of the previous pulse relaxed into the bulk material. This process continued for each pulse with its respective location $x_0$ and $y_0$, and post ablated mesh, until every pulse was simulated.

**Table 1:** Parameters used to model the laser-tissue interaction in Abaqus 2016a.

Parameter	Symbol	Value	Unit	Source
Density	$\rho$	2033	kg/m^3^	^53–59^
Thermal conductivity	*k*	0.54	W/mK	^51,54,56,58,59^
Specific heat capacity	*c*_*p*_	1.440	kJ/kgK	^53–55,58^
Beam radius $\left( 1/e^{2}\right)$	$\omega _0$	10	$\upmu \hbox {m}$	^33^
Maximum fluence *F*$(x = 0,\,y = 0,\,z = 0)$	*F*_*0*_	7.719	J/cm^2^	^33^
Threshold fluence *F*$(x = 0,\,y = r_{th},\,z = 0)$	*F*_*th*_	1.511	J/cm^2^	Eq. (1)
Threshold radius	*r*_*th*_	9.03	$\upmu \hbox {m}$	Eq. (1)
Pulse repetition frequency	PRF	1	kHz	^33^
Scan speed	*v*_*s*_	2	mm/s	^33^
Pulse duration (FWHM)	$\tau _p$	6	ps	^33^
Effective penetration	$\delta p_{eff}$	957	nm	Eq. (2)
Absorption coefficient	$\mu _a$	0.12	/mm	^49^
Reduced scattering coefficient	$\mu _s'$	2.45	/mm	^49^
Backscatter coefficient	*k*	5.02		^50^
Reflectivity	*R*	0.4		^49^
Ambient temperature	*T*_amb_	294.15	K	
Ambient heat sink coefficient	*h*	270	W/m^2^K	
Convection coefficient	*h*_a_	10	W/m^2^K	^52^

### Micropillar extraction directly by laser ablation

The use of focused ion beam milling for micropillar extraction has been preferred over laser ablation as it offers sub-micron precision while limiting the depth of impact on material composition to tens of nanometres^33–35^. The major disadvantage of focused ion beam milling, however, is the lack of throughput capacity required to rapidly screen micromechanical behaviour of tissue. Picosecond laser ablation has the potential to be a cost effective substitute capable of rapidly producing hundreds of test specimens under physiological conditions. To determine whether micropillars can be produced directly by picosecond laser ablation, an additional 298 pulses with consistent parameters were simulated from the final scanning diameter of 50 $\upmu {\text {m}}$ inward to 30 $\upmu {\text {m}}$.

The numerical modelling results representing Groetsch et al.^33^ inbound anticlockwise hatch pattern inward to a final diameter of 50 $\upmu {\text {m}}$ were verified by using post production compositional analysis, namely Raman microscopy and Energy Dispersive X-ray Microanalysis, to indicate whether the collagen and/or mineral phase of the MTLT samples were effected by the laser-tissue interaction.

### Raman microscopy

A confocal Raman microscope (inVia Renishaw, UK) was used to obtain Raman spectra prior to, and post laser ablation. Two samples were chosen for the ultramilled surface with a total of six measurements taken prior to laser ablation, and a single pre-pillar was chosen with three measurements taken post ablation. The following parameters were identified to show the best outcome with respect to the signal-to-noise ratio during the measurements: laser wavelength of $\lambda = 785$ nm (fixed by the set-up), ${\text{P}}\,_{laser}$ =  $1.163 \pm 0.321$ mW, four accumulations, wavenumber range of 200–2000 1/cm, exposure times of 120 s. An optical power meter was used prior to testing to ensure that all measurements had a similar power spectra.

**Figure 3:**
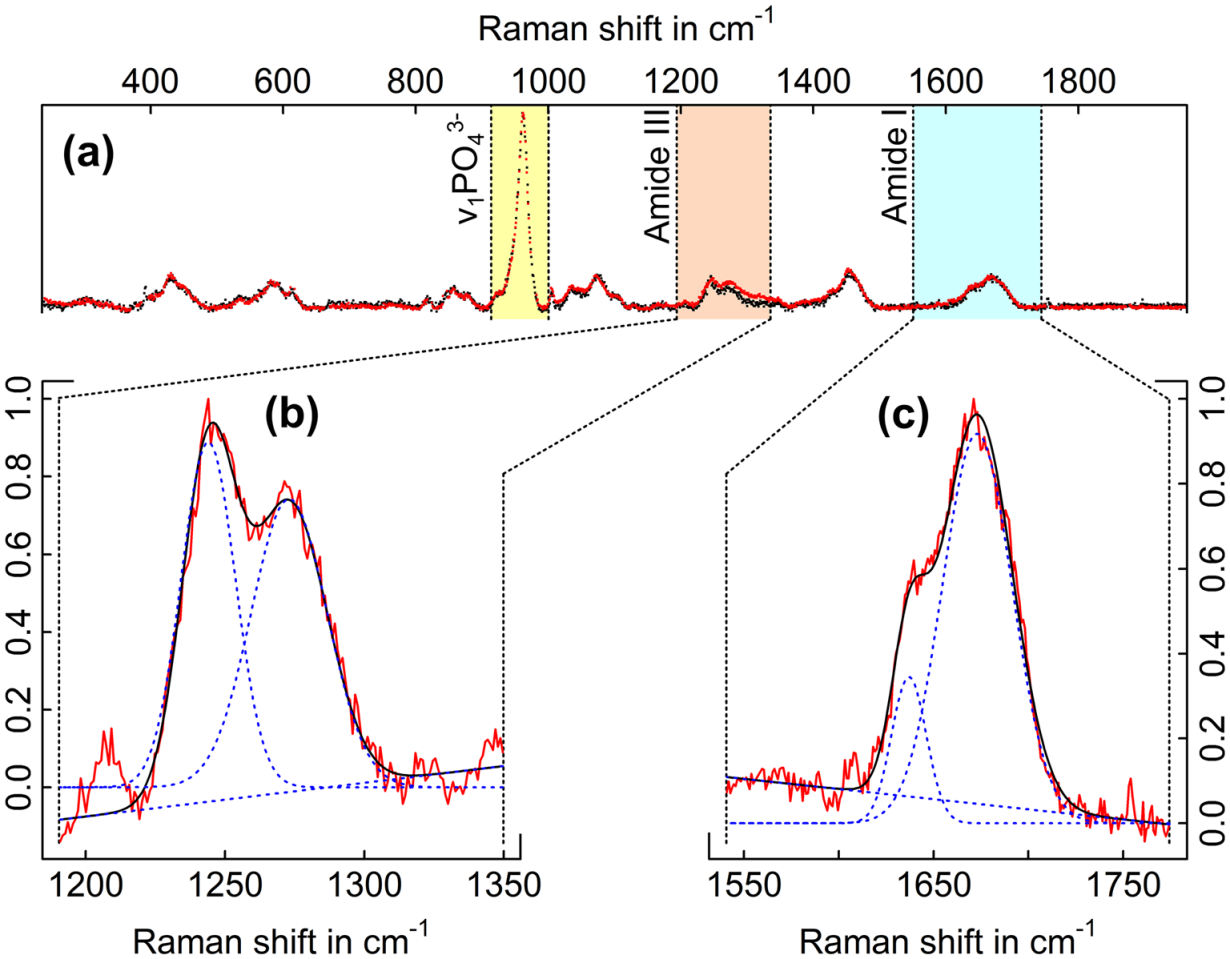
Raman spectral analysis. (**a**) Baseline corrected spectra of an ultramilled (black) and laser ablated pre-pillar (red) measurement. All y-axis values, intensity counts, are normalised in each of the graphs presented. (**b**) Gaussian fitting of the Amide III spectral response. (**c**) Gaussian fitting of the Amide I spectral response. Unsmoothed data was fitted with respect to each components given bands.

Figure 3a shows a baseline corrected Raman spectrum for an ultramilled sample^60^. We calculated the intensity ratios of I_1670_/I_1640_ and I_1245_/I_1270_ within the amide I (band 1616–1720 1/cm) and amide III (band 1190–1295 1/cm) peaks respectively as they are reportedly sensitive to denaturation^20^. Unal et al.^20^ observed significant increases in these ratios when thermally treating bovine cortical bone. Therefore, an increase in ratio post laser ablation would indicate denaturation. These ratios were determined by isolating the amide peaks by their given bands and fitting a two term Gaussian function to the unsmoothed data (Fig. 3) using^61^:

15 $$\begin{aligned} F(x) = \sum \limits _{i=1}^{2}I_i\,\exp \left[ -\left( \frac{x-P_i}{W_i}\right) ^2\right] \end{aligned}$$

here *I* denotes the Raman scattering intensity, *W* the full width at half maximum intensity, *P* the band’s peak position and *i* is the index of the constituent Gaussian curves. We also analysed the commonly reported mineral-to-matrix ratio of the main phosphate $v_1 PO_{4}^{3-}$ (band 910–1000 1/cm) and amide I peaks by means of numerical integration of the fitted Gaussian function^17,34,62,63^.

### Energy dispersive X-ray microanalysis (EDX)

Energy dispersive X-ray microanalysis^64,65^ was performed on the ultramilled and laser ablated pre-pillar samples. A Quanta FEG 650 SEM (FEI, Thermo Fisher Scientific, US) equipped with an Oxford Instruments X-maxN 150 EDX detector was used for the EDX measurements. Two ultramilled and four laser-ablated pre-pillar samples were selected with three spectra per sample being measured. An energy of ${E}$ = 10 keV was used such that it was twice the energy of any signal coming from relevant atomic elements in the sample. A scan window of 4.0 $\upmu {\text {m}}$ in horizontal direction and 3.5 $\upmu {\text {m}}$ in vertical direction was used to acquire the spectra over a lifetime of 10 ms for each measurement. Energy and lifetime was chosen to be small to avoid any beam damage during the acquisitions of the spectra^66,67^. The surface roughness of the different samples, corresponding to the subsequent preparation steps, was checked via 3D surface profiles by using focus variation microscopy (Alicona Infinite Focus, Austria)^40^.

Atomic elements were identified via a spectral analysis conducted in the XL30 Lab6 software. We analysed the chemical composition most relevant to bone tissue including the calcium (Ca) and phosphorus (P) peak, magnesium (Mg), sodium (Na), carbon (C) and oxygen (O). We used Ca/P ratio as a metric to determine physio-chemical change in the mineral phase post laser ablation as calcium and phosphorus constitute $\sim 30$ wt% of bone^68,69^.

### Statistical analysis

Statistical analyses were performed in R^70^ for measurement taken before and after the laser-tissue interaction. For the Raman measurements, a Wilcoxon rank sum test was used to compare differences in the amide intensity ratios. Here, a significance level of *p* = 0.05 was used. For the mineral-to-matrix ratio, a two-tailed *t*-test was used to identify, if any, a change. Normality was first verified by Shapiro–Wilke test (ultramilled, *p* = 0.82. laser ablated, *p* = 0.98). For the EDX measurements, a two-tailed *t*-test was conducted to determine any significant difference in the means of Ca/P ratio before and after the laser-tissue interaction. The XL30 Lab6 software simply outputted a mean and standard deviation after post processing, thus, we assume that the EDX measurements were normally distributed. Statistical significance level was *p* = 0.05.

## Results

### Micropillar temperature elevation

**Figure 4:**
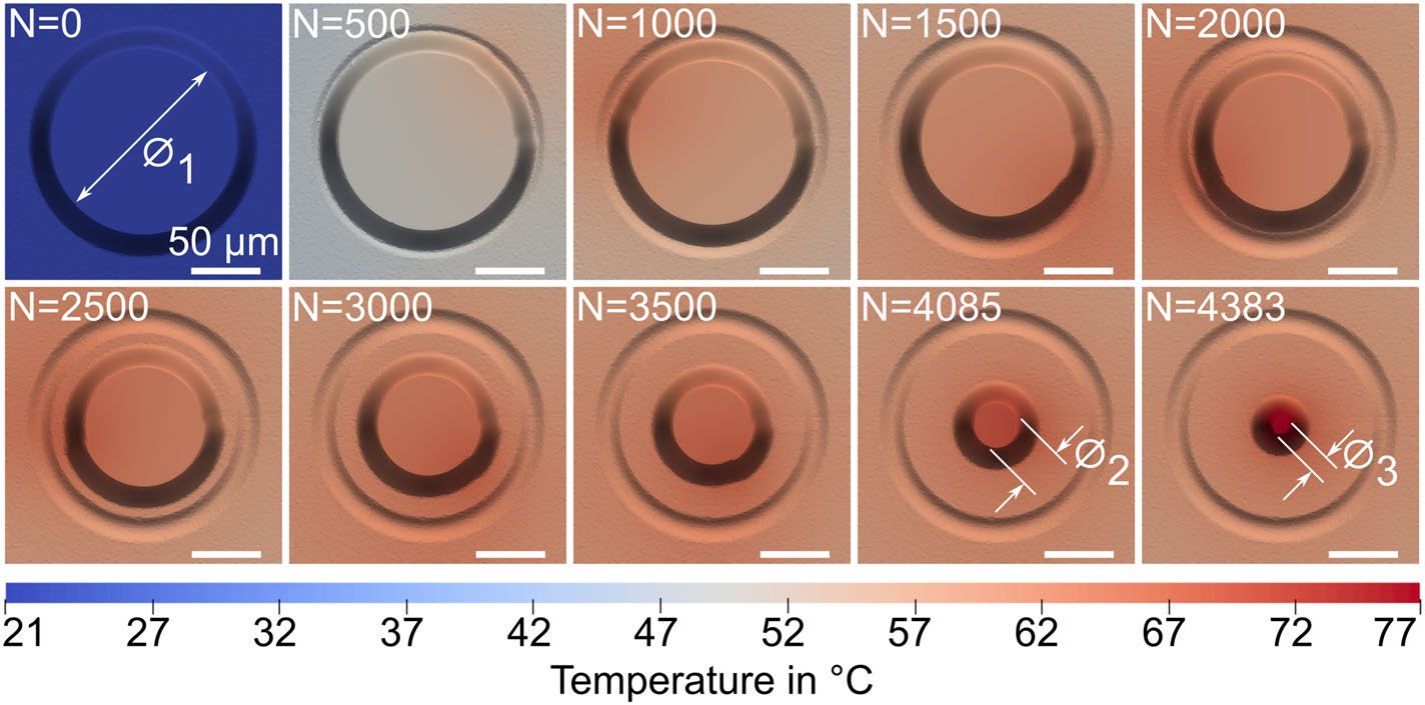
Plan view of the pre-pillar isolation. *N* is the pulse number in the scanning pattern. ${N}$ = 0, $\varnothing _1$ = 131.94 $\upmu {\text {m}}$ is the initial surface diameter at the start of the 150–50 $\upmu {\text {m}}$ inbound anticlockwise hatch pattern. ${N}$ = 4085, $\varnothing _2$ = 31.94 $\upmu {\text {m}}$ is the final pre-pillar that Groetsch et al.^33^ produced. ${N}$ = 4383, $\varnothing _3 = 11.94\;\upmu {\text {m}}$ is a theoretical pillar produced with consistent scanning parameters down to a diameter of 30 $\upmu {\text {m}}$.

All 4085 pulses that constitute the spiral inbound anticlockwise hatch pattern from a diameter of 150 $\upmu {\text {m}}$ inward to 50 $\upmu {\text {m}}$ were solved in Abaqus in 84 days (See Video S2 in “Supplementary Materials”). The pulse specific geometric evolution of the pre-pillar is presented in Fig. 4. The constant pulse separation distance of 2 $\upmu {\text {m}}$ resulted in a removal rate of $\sim 195$ $\upmu {\text {m}}$^3^ per pulse.

**Figure 5:**
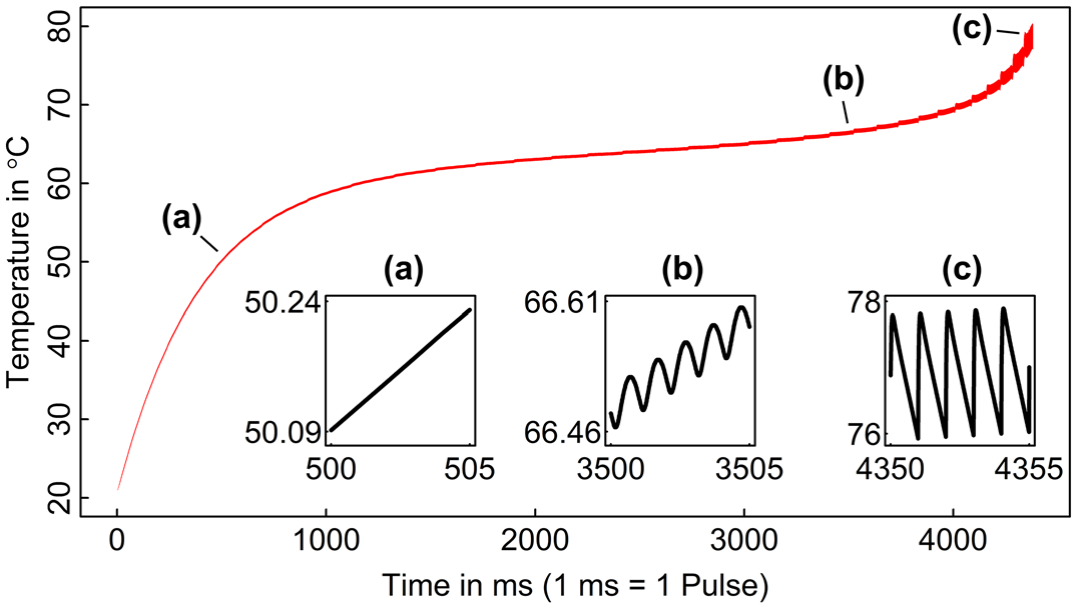
Temperature response of the micropillar. All 4383 pulses are presented. The main curve in red shows the variation of temperature in the micropillar with the upper and lower bounds corresponding maximum and minimum temperatures respectively. The sub plots show the temperature response at the centre of the micropillar, across a more sensitive time period where there is a development of localised heating as the dense temperature distribution of each pulse diffuses through the micropillar.

The temperature elevation within the $6.08\,\upmu {\text {m}}$ diameter, $12.44\,\upmu {\text {m}}$ height micropillar volume was evaluated throughout the 4.085 s laser scanning period. After all 4085 pulses, the maximum temperature in the micropillar was 70.58 $^{\circ }{\text {C}}$ (Fig. 5). This is 79.42 $^{\circ }{\text {C}}$ lower than that at which hydrogen bonded water is reported to evaporate in dehydrated collagen fibrils^26^. The result would suggest that the picosecond laser ablation regime did not elevate micropillar temperature to a magnitude that would induce denaturation of the constituent collagen fibrils. The sub-plots in Fig. 5 show the development of a pumping or oscillating temperature response within the micropillar. At smaller scanning radii, the beam axis is in close proximity to the micropillar, such that there is a noticeable local elevation in temperature as the pulse energy diffuses radially away from the beam axis.

Continuation of the inbound anticlockwise hatch pattern to a final radial pass of 15 $\upmu {\text {m}}$ resulted in a pillar with a surface diameter of 11.94 $\upmu {\text {m}}$ (Fig. 4) and taper angle of $12.14^{\circ }$. We evaluated the maximum temperature at any position within the 11.94 $\upmu {\text {m}}$ pillar. This maximum temperature was 81.69 $^{\circ }{\text {C}}$, again 68.31 $^{\circ }{\text {C}}$ lower than 150 $^{\circ }{\text {C}}$.

### Raman measurements to estimate the laser-tissue interaction effect

The comparison of the ultramilled surface and laser ablated pre-pillar samples suggests that the laser ablation regime had no impact on the physio-chemical composition of MTLT. No additional phases were present after the laser-tissue interaction and the Amide I and III intensity ratios, which are attributed to helical stability, showed no increase (Amide I, *p* = 1. Amide III, *p* = 1) (Table 2)^20^.There was no significant difference found in the mineral-to-matrix ratio before and after laser ablation (*p* = 0.86).

**Table 2:** Overview of Raman results for ultramilled surface and laser ablated pre-pillars including amide I and III intensity ratios and integrated areas mineral-to-matrix ratio. The values are presented as mean $\pm ~\text {SD}$.

Quantity	Ultramilled surface (*N* = 6)	Laser ablated pre-pillars (*N* = 3)
I_1670_/I_1640_	$1.552\pm 0.066$	$1.461\pm 0.007$
I_1245_/I_1270_	$1.251\pm 0.059$	$1.125\pm 0.018$
${\text {A}}_{v_1 PO_{4}^{3-}}$/A_Amide I_	$2.581\pm 0.146$	$2.587\pm 0.027$

### EDX measurements to estimate the laser-tissue interaction effect

The main prominent peaks of Ca, C, O, P, Mg and Na were identified for all samples and no additional atomic elements were detected after laser ablation. No significant difference (*p* = 0.58) was found for the mean values of Ca/P ratios, which were $1.73 \pm 0.15$ for ultramilled samples and $1.76 \pm 0.17$ for laser ablated pre-pillars. It confirms that the laser-tissue interaction did not effect the Ca/P ratio which we use as an indication of the mineral phase quality. Sample surface roughness were comparable for the different preparation steps and, thus, had no influence on the inter-sample comparison with respect to the EDX results.

## Discussion

We have established a finite element modelling technique allowing us to assess the temperature response of laser ablated tissue samples. We used experimental laser parameters from a micropillar extraction protocol for mineralised collagen fibres reported by Groetsch et al.^33^. The modelling framework also allows for the interchangeability of tissue properties and scanning patterns. The simulation of the 4085 pulses that constitute the final spiral inbound anticlockwise hatch pattern from a diameter of 150 $\upmu {\text {m}}$ inward to 50 $\upmu {\text {m}}$ has shown that the induced temperature elevation within the micropillar region remains lower than half of the 150 $^{\circ }{\text {C}}$ denaturation onset temperature^26^ throughout the 4.085 s laser scanning period. The modelling results dictate that the used laser protocol is safe with regards to temperature exposure, enabling us to confidently and rapidly produce hundreds of micropillars for use in high throughput mechanical testing.

We developed a pulse specific geometric profile of the MTLT prior to heat transfer analysis. Here, an effective penetration depth of 957 nm was calculated such that an ablated volume could be removed from a surface profile representative of the MTLT after each successive pulse in the scanning pattern. This was done through interpretation of the post ablated geometry to first determine a threshold fluence (1.511 J/cm^2^), which, though for different incident wavelengths falls within published values for cortical bone of 0.78–3.29 J/cm^2^^32,69,71,72^. The employment of these interpreted parameters yielded a surface profile with consistent dimensions to the pre-pillars produced by Groetsch et al.^33^ (Fig. 1e).

The simplification of the two temperature model (Eqs. 6–9) into a single generalised heat equation by modelling the condition where $\left( T_e = T_l = T\right)$ allows for a reduction of the finite element model to a single mesh domain, with the assigned bulk thermophysical properties of bone. Modelling the two heat equations would require two geometrically identical mesh domains that exchange energy, with the first representing the electron domain and the second representing the lattice (see e.g.^48^). The simplification allowed us to mitigate the lack of data surrounding free electron heating and relaxation with regards to 515 nm irradiation of bone. This simplified approach implies an idealised heat addition where the temperature response of the bulk is spatially equal to that of the energy absorption rate. Inclusion of electron conductivity would see electron energy diffusion prior to lattice heating. The pulse energy deposited into the system remains the same regardless of the made simplification, and the thermalised temperature is comparable as the bulk heat capacity is given as $C = C_l + C_e$^48^. This comparable thermalised temperature was a further motive for the simplification as the scanning pattern time frame is in the order of 10^9^ longer than the thermalisation time of a picosecond pulse.

The major components of the mineralised collagen fibre; carbonated hydroxyapatite, water and collagen all exhibit band gaps larger than the photon energy delivered by a single 515 nm photon^73–75^. We have not investigated the phase change mechanism within the ablated volume. However, it can be assumed that the intensity is sufficient to induce non-linear optical processes needed to generate a free electron plasma, such as multiphoton and impact ionisation^12,46^. This free electron generation influences the optical properties such that further penetration and energy deposition is confined to a thin layer proximal to the target surface^10,76^. The subsequent ablated volume exhibits a low aspect ratio^13^. Our interpretation of an effective penetration depth of 957 nm led to an ablated volume with a characteristically low aspect ratio in line with that of dielectric material ablation. By definition, the laser-tissue interaction of the major components of the mineralised collagen fibre would dictate that there is no electron absorption of 515 nm light at low intensities. We, however, selected an effective attenuation and back-scatter coefficient derived from Monte–Carlo simulations of light transport in tissue^49,50^ to mitigate the lack of data needed to characterise the temporal optical properties of bone during high intensity 515 nm wavelength irradiation. The used parameters selected from Ugryumova et al.^49^ were calculated from measurements of diffuse reflectance of a white light source as opposed to a laser source. The numerical values derived from a laser interaction could differ from the used modelling parameters. The increase in temperature across the scanning period may further influence these properties. For example, if the influence of temperature increased reflectivity, the deposited energy within the MTLT would decrease, ultimately reducing the final temperature within the micropillar. We chose these values such that the modelling parameters where informed by the best available experimental data. The application of a linear attenuation coefficient infers there is a density of free electrons present in the MTLT prior to the laser-tissue interaction to mediate electron heating. The made assumptions could have led to an under estimation of the temperature response in the micropillar. A small increase in temperature response induced by single pulse, however, would see a large increase in temperature across the entire scanning pattern due to the accumulation of thousands of pulses. This large increase would have been evident in the observed Raman spectra due to the sensitive nature of collagen with respect to temperature exposure. We would have observed noticeable changes in the spectrum of a pre-pillar observation had the temperature exposure been in excess of a denaturation threshold. As we did not observe this, we deem our modelling parameters and simplification just and reasonable.

The acquired Raman spectra for the ultramilled surface and laser ablated pre-pillars show all characteristic Raman peaks reported for bone tissue^17,34,63^. No additional phases were formed as a result of laser ablation. The mineral-to-matrix ratio has been reported to correlate with the mechanical properties of bone at both macroscopic and micrometre length scales as well as being an indicator for disease classification^77–79^. If the mineral composition changed post laser ablation, the in situ testing results might not reflect the true mechanical properties of the unprocessed MTLT. We have shown that the ratio of integrated areas of the main phosphate and amide I (${\text {A}}_{v_1 PO_{4}^{3-}}$/A_Amide I_) peaks did not change post laser ablation. The EDX measurements also showed no significant difference in the calcium-to-phosphorus (Ca/P) ratio post laser ablation. Though not directly quantifiable, the Ca/P ratio can give an indication of temperature exposure. Here, changes can be attributed to the formation of different products, for example, pyrophosphate Ca_2_P_2_O_7_ can form between 200 $^{\circ }{\text {C}}$ and 400 $^{\circ }{\text {C}}$^80^. We can infer from the Raman and EDX results that mechanical testing by Groetsch et al.^33^ should best represent dehydrated conditions as the material composition with respect to the mineral content was unchanged when samples were prepared by picosecond laser ablation.

Fourier transform infrared spectroscopy studies have used the ratio I_1660_/I_1690_ that constitutes the main amide I peak as a metric to qualitatively assess denaturation, where the ratio can be associated to the ratio of nonreducible/reducible collagen cross-links^81–83^. This ratio is sensitive to the excitation wavelength^84^ and we have not used this metric as no prominent shoulder to the amide I peak at $\sim 1690$ 1/cm was observed in any measurement. In place of this, we used Unal et al.^20^ reported ratios of I_1670_/I_1640_ and I_1245_/I_1270_, where an increased ratio is said to represent a loss of helical structure in mineralised collagen fibrils. The fitted amide curves and subsequent intensity ratios showed no significant increase, indicating that no denaturation occurred.

The compositional analysis indicates that the MTLT was unaffected by the laser-tissue interaction, however, care must be taken when interpreting the results due the small sample sizes. Larger sample sizes were unattainable as the measurements were taken prior to this study as part of Groetsch et al.^41^ observations that focused on differences between structured and mechanically deformed micropillars and not on the ultramilled and laser ablated surfaces. Additionally, the ratios presented are not a direct quantifiable measure of denaturation or mineral quality. They do, however, allow us to qualitatively asses relative changes in matrix qualities and the lack of change post ablation does ultimately suggest that the collagen and mineral phases of the pre-pillar are not affected by the laser-tissue interaction. Further to this, Canteli et al.^85^ analysed spectra for laser ablated bovine cortical bone that was thermally damaged and observed two strong bands at $\sim 1350$ 1/cm and $\sim 1580$ 1/cm. These peaks were associated with the presence of amorphous carbon within the material as a result of carbonisation. None of our samples exhibited these peaks, and there was no visible evidence of carbonisation.

The used absorption and scattering coefficients were taken from a study which tested equine cortical bone samples immersed in saline solution^49^, which, is more representative of the physiological conditions found in clinical laser procedures. These parameters were chosen to mitigate the lack of data for dry bone samples irradiated by 515 nm wavelength light. Groetsch et al.^33^ fabricated micropillars from air dried samples, however, the mineralisation, which has a dominant influence on optical properties^49^, is independent of hydration state. Therefore, we believe our results are indicative. Moreover, we believe the results can be applied to physiological conditions, where the induced temperature of the micropillar is above reported denaturation threshold temperatures for hydrated collagen ($\sim 54\;^{\circ }{\text {C}}$^25,28^). Micropillar extraction is a unique internal heat transfer case dissimilar to the external case found in orthopaedic hole drilling. During micropillar extraction, the pillar itself is the central axis of any scanning pattern, allowing for unperturbed heat diffusion through the pillar which must remain unaffected. Whereas, in the instance of hole drilling, the hole itself limits heat diffusion to material on the hole circumference that is opposite to the pulse location, reducing the temperature of all surrounding tissue. Our case, therefore, represents a worst case scenario of heat coupling into the material. In any case, simply increasing the scanning distance from critical structures such as nerve tissue could accommodate this increased temperatures. Our modelling protocol allows for optimisation of scanning patterns to limit the thermal damage of sensitive tissue. In addition, it must be noted that Chen et al.^23^ observed for isothermal exposure testing at 65 $^{\circ }{\text {C}}$, that collagen fibrils show low rate, reversible shrinkage for $\sim 15$ s. Our results stipulate that Groetsch et al.^33^ protocol could safely fabricate 72 $\upmu {\text {m}}$ pillars in 3 s under physiological conditions prior to the micropillar temperature reaching 65 $^{\circ }{\text {C}}$.

Under dehydrated conditions, the theoretical continuation of the inbound anticlockwise hatch pattern inward to a final 30 $\upmu {\text {m}}$ diameter pass did not excessively heat the resultant 11.94 $\upmu {\text {m}}$ diameter pillar. Even when laser parameters achieve optical breakdown of the target tissue, a finite nanometre skin layer proximal to the ablated volume may be elevated to temperatures in excess of tissue damage thresholds. The volume of damaged tissue, which will be localised to the surface, would be several orders of magnitude smaller than that of the micropillar. Therefore, this would not compromise their integrity. Furthermore, Plötz et al.^86^ ablated bovine cortical bone using an 8 ps, 1,064 nm wavelength laser and when operating at fluences slightly above threshold, did not observe any damage by means of histological analysis at $40 \times$ magnification. The process parameters used by Groetsch et al.^33^ were similarly optimised to operate slightly above threshold fluence. This combined with the lack of recast when observed under SEM, reinforces our assertion of a non-thermal ablation mechanism.

Though not the focus of this paper, it is important to consider the geometry that can be achieved by laser ablation. Vertically orientated pillars are desirable as when compressed, they induce a quasi-homogeneous stress state from which material properties can be directly obtained^33–35^. The pillar we present, however, exhibits a taper angle of 12.14$^{\circ }$ (Fig. 1e). This geometric constraint must be considered either by resolving testing results with respect to geometry with tools such as finite element modelling or removing the taper angle prior to testing. Lim et al.^32^ have demonstrated that vertically orientated pillars can be produced directly by laser ablation and our results verify that it is reasonable to further investigate laser ablation as an extraction technique where this achievable geometry can now be the focus.

We recommend, based on the presented results, that micropillars can be extracted directly by ultrashort pulsed laser ablation without altering the physio-chemical and structural composition of the constituent mineralised collagen fibres. This was done through modelling and extrapolation of the laser-tissue interaction of a used extraction protocol. The simulated temperature response within the micropillar is below that at which conformational change within the collagen fibrils is reported to occur. Further to this, post-production compositional analysis showed no relative changes post laser ablation with respect to biomarkers that are attributed to collagen denaturation. The results will allow us to rapidly screen large numbers of samples to further understand the influences that bone diseases have on the micro- and nanoscale mechanical behaviour of bone tissue.

## Supplementary Information

Supplementary Information.

Supplementary Video S1.

Supplementary Video S2.
