# Supplementary material for: Heat impact during laser ablation extraction of mineralised tissue micropillars
Source: Sci Rep. 2021 May 26;11:11007. doi: 10.1038/s41598-021-89181-9 (PMC8155055; doi:10.1038/s41598-021-89181-9)
Supplement: Supplementary file 1 — Supplementary Information. [file 41598_2021_89181_MOESM1_ESM.docx]

Supplementary material to:

Heat impact during laser ablation extraction of mineralised tissue micropillars

Samuel McPhee^a^, Alexander Groetsch^a^, Jonathan D. Shephard^b^, Uwe Wolfram^a,^*

^a^ Institute of Mechanical, Process and Energy Engineering, School of Engineering and Physical Sciences, Heriot-Watt University, Edinburgh, UK

^b^ Institute of Photonics and Quantum Sciences, School of Engineering and Physical Sciences, Heriot-Watt University, Edinburgh, UK

* Corresponding author, Email address: u.wolfram@hw.ac.uk (Uwe Wolfram)





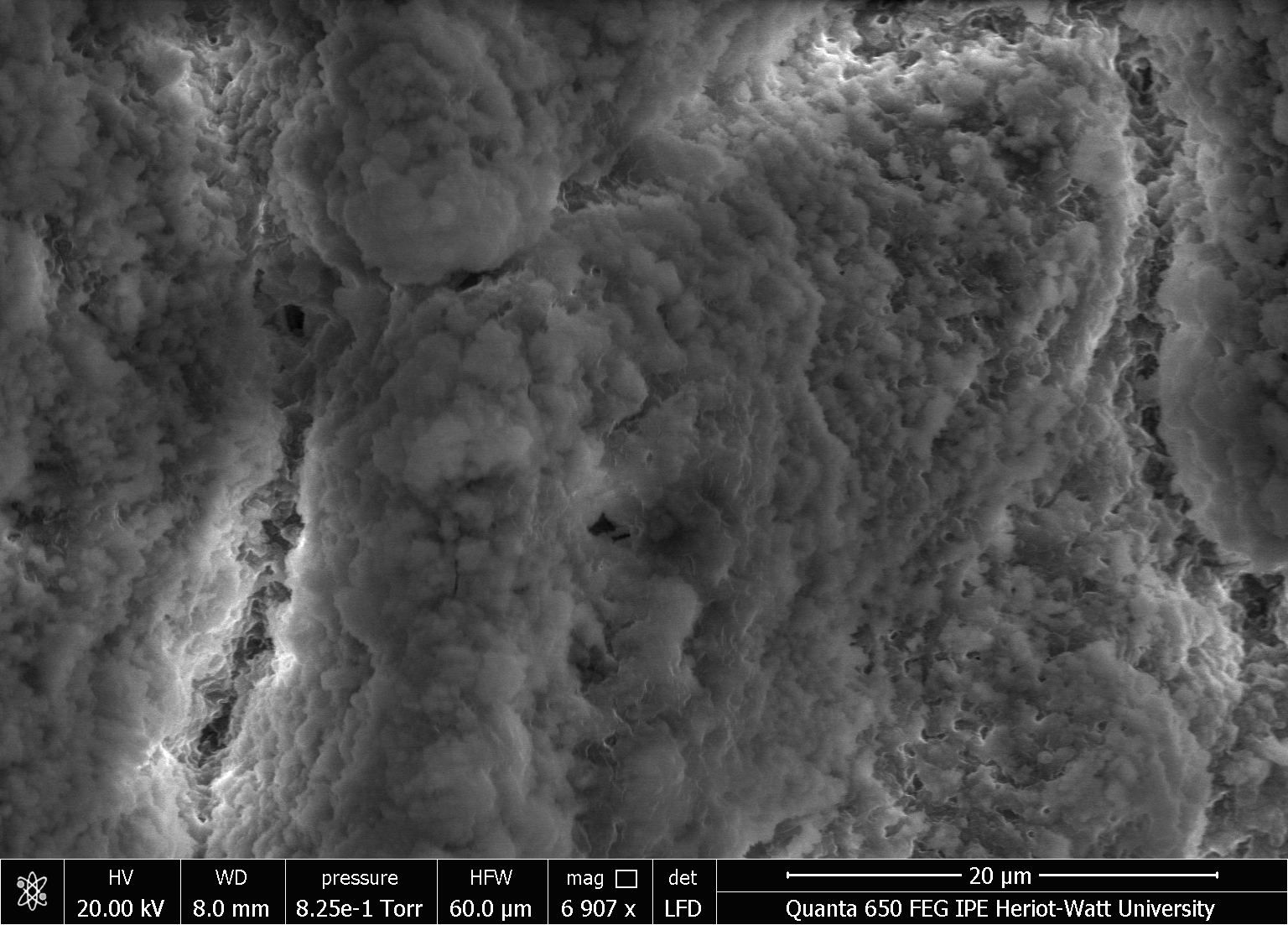





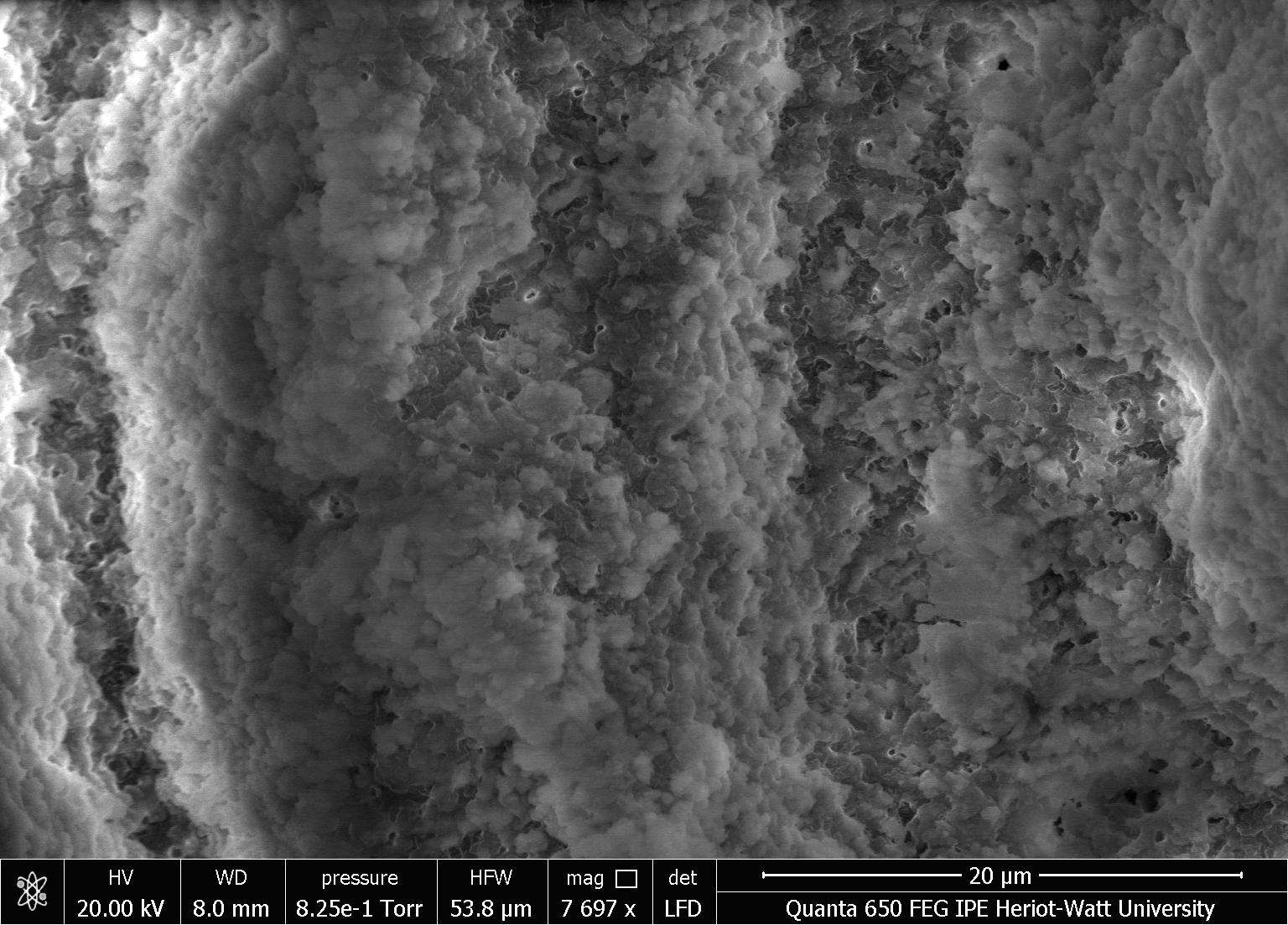


**Figure S1: SEM images of 515 nm, 6 ps laser ablated mineralised turkey leg tendon.** The morphology does not exhibit a high degree of smoothing expected from melting and no recast in the form of globular deposits are observed. This indicates that the chosen process parameters achieve a non-thermal ablation mechanism.

**Video S1: Energy absorption rate.** This video provides visualisation of the temporal and spatial aspects of the energy absorption rate described in the methods section. (File uploaded separately)

**Video S2: FEA simulation.** This video visualises the numerical modelling results of all 4383 simulated pulses that constitute the inbound anticlockwise hatch pattern from a diameter 150 µm inward to 30 µm. (File uploaded separately)
